# Cervico-Facial Actinomycosis

**Published:** 1907-10-12

**Authors:** 


### Illustrative Cases.

### CERVICO-FACIAL ACTINOMYCOSIS.

The following case teaches at least three points about actinomycosis in man, namely (1) the comparative painlessness of even an extensive lesion in the skin; (2) the chronicity of the trouble; and (3) the inadvisability of relying on iodide of potassium alone if cure is to be effected. The iodide certainly relieves the condition to some extent, but it is by no means the specific remedy that it is in the case of gummata. If actinomycosis is diagnosed early enough, radical treatment by surgical means should at once be resorted to in addition to potassium iodide; otherwise the skin lesion may become as difficult as lupus vulgaris to eradicate.

A well-grown, highly intellectual man presented himself for medical examination in September 1900, complaining of a swelling of the right cheek which had begun insidiously five weeks before; he also complained of some trismus, and of pain in a right superior molar tooth, which was movable in its socket. It appeared that he had never been in the habit of holding straw in his mouth, and his only connection with straw was that he not infrequently lay down on some to take a midday nap. He was not a worker amongst horses. The swelling had gradually increased, and several abscesses had formed, each of which had opened spontaneously without any marked inflammatory symptoms, and without any pain to speak of. The whole of the right side of his face was found to be swelled, and there was an extensive diffuse hard patch, which was subcutaneous, adherent to the deeper structures, and extended from the right temple down over the right masseteric region on to the right submaxillary region. The masseter was contracted to such a degree that it was difficult to insert a finger into the mouth. The skin over the central part of the indurated patch was reddened and adherent, and beneath it four separate masses could be felt, each the size of a small nut, and on pressing these a grumous pus mixed with blood could be expressed; on careful examination this pus was noticed to contain a great number of minute yellowish granules. Microscopical examination of these showed typical actinomyces, and the pus was also teeming with staphylococci. There was a small gland to be felt in the left submaxillary region, but on the right side, where the trouble was, no involvement of the lymphatic glands could be made out. There was a very remarkable absence of pain; even when the swelling was sufficient to nearly close the right eye, there was almost no pain at all. The one loose tooth having been removed, the rest were all noted as being perfectly sound and healthy. Treatment was by the oral administration of potassium iodide ℨss. quotid, by laying the abscesses more freely open with the knife, and by an antiseptic dressing.

By October 10, 1900, there was little or no alteration in the condition; three fluctuating and fistulous abscesses, freely discharging pus, were still draining. On October 31 the mouth could be opened a little further than before. One of the abscesses in the temporal region had ceased to discharge, and within it yellowish red granulations were forming and projecting from its orifice. Two fresh abscesses had formed on the cheek; these were incised and drained, yielding first a yellowish caseous material, and later pus and blood; the discharge of the pus was much interfered with by the rapid formation within the cavities of interlacing networks of granulation tissue. The iodide was increased to ℨjss quotid. By December 1900 the patches of hard infiltration below the jaw had resolved without suppuration, but the skin over the right side of the face was still red and tense; in the region of the malar bone there were two fistulous openings which led into devious ramifications beneath the skin; little pus was coming from the sinuses, but inside them there were extensive fungous granulations. There was nowhere any necrosed bone to be detected, but the indurated subcutaneous tissues could not be moved upon the underlying bones. The abscess that had been present in the temporal region was healed, the scar being red, irregular, and fixed. Two fresh subcutaneous nodules formed towards the end of December, each the size of a large bean, one situated deeply in the cheek, the other above the zygomatic process; notwithstanding the continued administration of ℨjss of potassium iodide daily, the upper of these two nodosities softened and broke down, discharging actinomycotic caseous material and pus as the others had done. The other nodule disappeared slowly without surgical intervention.

He was lost sight of for six years, coming under observation again in June 1906. The lesions had never completely healed throughout all this time; sometimes they had scabbed over, but slight discharge soon recurred; the patient had not taken any iodide during all this time, and had been content to put up with the trouble without any particular treatment. When now seen much of the skin and subcutaneous tissues which had been indurated and red six years before had become supple, soft, and white again; the scars of former abscesses were deep, white, and slightly depressed below the surrounding surface. Near the angle of the jaw between the latter and the lobule of the ear there was a diffuse soft recent swelling, ill-defined, subcutaneous, and not fixed to the deeper structures; and behind this there were several small superficial ulcers in the skin, covered with dry crusts. Iodide of potassium was again administered in large doses, and the swelling rapidly began to heal up. The deformity and disfiguration of the right side of the face and neck are very considerable; the scarring is not dissimilar to that which results from the healing of gummata; the actinomycotic lumps develop without any apparent relationship to the general health of the patient, and every time they reappear one is struck by the remarkable slightness of the pain in comparison with what would be the case were similar swelling and abscess formation due to any of the ordinary pyogenic organisms.

